# Influence of radiation dose and predicted tumor invasion depth on local recurrence after definitive chemoradiotherapy for stage 0–I esophageal squamous cell carcinoma: a propensity score-weighted, retrospective, observational study

**DOI:** 10.1186/s12885-022-09418-2

**Published:** 2022-03-21

**Authors:** Toshiki Ikawa, Ryu Ishihara, Katsunori Matsueda, Koji Konishi, Sachiko Yamamoto, Masahiro Morimoto, Naoyuki Kanayama, Teruki Teshima

**Affiliations:** 1 Department of Radiation Oncology, Osaka International Cancer Institute, 3-1-69 Otemae, Chuo-ku, Osaka-city, Osaka 541-8567 Japan; 2 Department of Gastrointestinal Oncology, Osaka International Cancer Institute, Osaka, Japan; 3 Present Address: Osaka Heavy Ion Therapy Center, Osaka, Japan

**Keywords:** Chemoradiotherapy, Dose-response relationship, Endoscopic mucosal resection, Esophageal neoplasms, Propensity score

## Abstract

**Background:**

The optimal radiation dose for treating non-metastatic superficial esophageal squamous cell carcinoma is unknown. In this retrospective observational study, we investigated the influence of radiation dose and pretreatment endoscopic prediction of tumor invasion depth on local recurrence after definitive chemoradiotherapy in patients with superficial esophageal squamous cell carcinoma.

**Methods:**

We analyzed 134 patients with clinical Tis–T1N0M0 esophageal squamous cell carcinoma who underwent chemoradiotherapy at our institution between 2006 and 2019. Patients were grouped into standard-dose (50.0–50.4 Gy) and high-dose (60.0 Gy) radiotherapy groups. The outcomes of interest were local recurrence and major local recurrence (endoscopically unresectable local recurrent tumors). Kaplan–Meier analysis and the log-rank test were used with propensity score and inverse probability of treatment weighting. Cox proportional hazards analysis was performed to identify predictors of local recurrence and major local recurrence.

**Results:**

The median follow-up times were 52 and 84 months for the standard-dose and high-dose groups, respectively. The adjusted 3-year local recurrence and major local recurrence rates in the standard-dose and high-dose groups were 33.8 and 9.6% (adjusted hazard ratio, 4.00 [95% confidence interval: 1.64–9.73]; adjusted log-rank *p* = 0.001) and 12.5 and 4.7% (adjusted hazard ratio, 3.13 [95% confidence interval: 0.91–10.81]; adjusted log-rank *p* = 0.098), respectively. Cox proportional hazards analysis showed that standard-dose radiotherapy and endoscopic findings of deep submucosal invasion are independently associated with local recurrence and major local recurrence.

**Conclusions:**

High-dose radiotherapy is more beneficial for local tumor control than standard-dose radiotherapy in patients with non-metastatic superficial esophageal squamous cell carcinoma. The use of high-dose radiotherapy may merit consideration for tumors with deep submucosal invasion.

**Supplementary Information:**

The online version contains supplementary material available at 10.1186/s12885-022-09418-2.

## Background

Superficial esophageal squamous cell carcinoma (SESCC) is defined as esophageal squamous cell carcinoma with the depth of invasion (DOI) limited to the mucosa or submucosa, regardless of lymph node or distant organ metastasis [1, 2]. Definitive concurrent chemoradiotherapy, as well as surgery and endoscopic resection, are the treatments of choice for non-metastatic SESCC [3]. As small mucosal tumors can be resected by endoscopic resection, chemoradiotherapy and surgery are generally used for large mucosal tumors or submucosal tumors. The survival rate in patients with non-metastatic SESCC after definitive chemoradiotherapy is reported to be comparable to that after esophagectomy [4–6]. However, residual tumors or local recurrence (LR) after definitive chemoradiotherapy can occasionally cause problems. The Japan Clinical Oncology Group 9708 study—a phase II trial to evaluate the efficacy and toxicity of definitive chemoradiotherapy using a 60.0-Gy dose for non-metastatic SESCC—reported that five of 72 (6.9%) patients experienced LR that could not be eliminated by endoscopic resection [6].

The prescribed dose is an important factor for local tumor control in radiotherapy, and the optimal dose has often been debated. The INT 0123 trial (Radiation Therapy Oncology Group 94–05) [7] revealed no benefits of high-dose radiotherapy (64.8 Gy) on local/regional control and survival in patients with early and locally advanced esophageal cancer. Based on these results, the standard dose of definitive chemoradiotherapy for esophageal cancer is considered to be 50.4 Gy. However, this study included only 15 patients with clinical T1 esophageal cancer, and the optimal dose for patients with non-metastatic SESCC has not been investigated. Compared to patients with locally advanced esophageal cancer, those with non-metastatic SESCC have a lower risk of developing lymph node and distant metastatic recurrence after definitive chemoradiotherapy, and local failure may directly lead to poor outcomes. The optimal dose in patients with non-metastatic SESCC may differ from that in patients with locally advanced esophageal cancer. Therefore, we conducted a retrospective observational study to investigate the effect of radiation dose on local tumor control in patients with non-metastatic SESCC. Recent advancements in endoscopic imaging have enabled more precise estimation of the DOI of SESCC [8]; therefore, we also investigated the association between the predicted DOI and local tumor control.

## Methods

### Patients

We retrospectively examined 179 patients with histologically proven, clinical Tis–T1N0M0 (International Union Against Cancer Tumor–Node–Metastasis classification [seventh edition]) esophageal squamous cell carcinoma who received definitive concurrent chemoradiotherapy at our institution between 2006 and 2019. We excluded patients with a follow-up time of < 3 months (*n* = 5), those with a history of surgery for esophageal cancer (*n* = 2), those who did not receive a sufficient radiation dose (> 40.0 Gy [*n* = 1]), those who did not receive concurrent chemotherapy (*n* = 20) or received concurrent chemotherapy for synchronous cancers in other organs (e.g., head and neck cancer [*n* = 5]), and those who received new combination chemotherapy in prospective clinical trials (*n* = 12). In total, 134 patients met the inclusion criteria and were enrolled in the study. All procedures performed in studies involving human participants were in accordance with the ethical standards of the institutional and/or national research committee and with the 1964 Declaration of Helsinki and its later amendments or comparable ethical standards. The study design was approved by the ethics committee of Osaka International Cancer Institute (approval number 20096). All patients provided written informed consent for the use of their data in clinical research before the administration of radiotherapy and had the opportunity to opt out of the study.

### Pretreatment evaluations

Pretreatment staging was based on gastrointestinal endoscopy and computed tomography of the neck, chest, and abdomen. Endoscopic staging of tumor invasion depth typically consisted of conventional imaging and magnifying narrow-band imaging (the details of endoscopic staging have been described previously [9]). Endoscopic ultrasonography was performed when endoscopy suggested that the tumor had invaded the muscularis mucosa or deeper. The clinical DOI was divided into three categories based on pretreatment endoscopic findings: EP/LPM (tumors limited to the epithelium or invading the lamina propria mucosa), MM/SM1 (tumors invading the muscularis mucosa or the submucosa to a depth of ≤ 200 μm from the lower border of the muscularis mucosa), and SM2 (tumors invading the submucosa to a depth of > 200 μm) [1, 2].

### Chemoradiotherapy

Patients were treated using linear accelerator-based external beam radiotherapy with three-dimensional conformal radiotherapy or intensity-modulated radiotherapy/volumetric modulated arc therapy (IMRT/VMAT) techniques. Before computed tomography simulation, radio-opaque clips were endoscopically placed at the cranial and caudal ends of the tumor. For treatments administered before March 2011, the clinical target volume (CTV) for the primary tumor included the esophagus between the clips with longitudinal margins of 20–30 mm, and the prescribed radiation dose was 60.0 Gy in 30 fractions. Since March 2011, the CTV for the primary tumor has included the esophagus between the clips with longitudinal margins of 5 mm, and the total dose has been de-escalated to 50.0–50.4 Gy in 25–28 fractions. However, increasing the total dose to 60.0 Gy was allowed at the radiation oncologist’s discretion. Elective nodal irradiation (ENI) was not routinely used before March 2011. Thereafter, ENI has been used primarily for clinical MM/SM1 or SM2 tumors. The CTV for ENI was determined based on tumor location (the details of the CTV for ENI have been described previously [10]), and the ENI dose was 40.0–41.4 Gy in 20–23 fractions. Concurrent chemotherapy typically consisted of cisplatin (70 mg/m^2^/day on Days 1 and 29) and 5-fluorouracil (700 mg/m^2^/day as a continuous infusion on Days 1–4 and 29–32). However, the dose was reduced, or other regimens were used, if necessary, based on the patient’s condition (other chemotherapy regimens are described in Additional file 1).

### Patient follow-up and evaluation of outcomes

Follow-up examinations were performed 1–2 months after chemoradiotherapy and then every 3–6 months for the first 2 years and every 6 months thereafter. Each examination consisted of gastrointestinal endoscopy and computed tomography of the neck, chest, and abdomen. The outcomes of interest in this study were LR and major local recurrence (MLR) after chemoradiotherapy. LR was defined as the persistence or recurrence of the tumor at the same esophageal level measured from the incisors and diagnosed using endoscopic biopsy confirmation of carcinoma. New lesions at the same level after 5 years following chemoradiotherapy or at another level were not considered LR. We also assessed MLR, defined as LR that was considered unresectable by endoscopic resection (endoscopic mucosal resection or endoscopic submucosal resection). LR requiring photodynamic therapy was regarded as MLR. To assess the effect of radiation dose on LR and MLR, we grouped patients into standard-dose (planned total dose of 50.0–50.4 Gy) and high-dose (planned total dose of 60.0 Gy) groups. To assess the effect of radiation dose according to the clinical DOI, we further divided patients into three DOI groups based on pretreatment endoscopic staging: EP/LPM, MM/SM1, and SM2 subgroups.

### Statistical analyses

First, we assessed the differences in baseline characteristics between the two dose groups using the Mann–Whitney *U* test for continuous variables and Fisher’s exact test or chi-squared test for categorical variables.

Second, we performed propensity score (PS) analysis using inverse probability of treatment weighting (IPTW) to account for the imbalance in baseline covariates between the two groups. PSs were estimated using covariates in the multivariable logistic regression analysis and plotted as histograms. Covariates included age (< 65, ≥ 65 and < 75, ≥ 75 years), sex (male, female), tumor location (cervical/upper thoracic esophagus, middle thoracic esophagus, lower thoracic esophagus), tumor length (≤ 40, > 40 and ≤ 80, > 80 mm), clinical DOI (EP/LPM, MM/SM1, SM2), and chemotherapy regimen (cisplatin and 5-fluorouracil, others). Receiver operating characteristic (ROC) curve and concordance statistic (c-statistic) analyses were used to test the appropriateness of the model. Covariate balance was assessed using standardized mean differences [11]. A standardized mean difference of < 0.10 for a given covariate was considered as an acceptable balance. We performed Kaplan–Meier analysis and the log-rank test with IPTW adjustment [12] to assess differences in LR and MLR between the two dose groups and estimated the corresponding hazard ratios (HRs). LR and MLR were measured from the start of chemoradiotherapy, and patients were censored at the date of last follow-up or death. We also performed unadjusted Kaplan–Meier analyses stratified by clinical DOI to examine its effect on differences in LR and MLR between the two dose groups.

Third, we performed univariable and multivariable analyses using a Cox regression model to analyze the associations of the two dose groups and clinical DOI with LR and MLR and determine the estimated HRs. Covariates with *p*-values < 0.02 in the univariable analysis were included in the multivariable analysis.

All statistical tests were two-sided, and a *p*-value < 0.05 was considered statistically significant. All analyses were performed using R (version 3.6.3) (R Foundation for Statistical Computing, Vienna, Austria) and SAS (version 9.4) (SAS Institute Inc., Cary, NC, USA).

## Results

### Patient characteristics and propensity score weighting

Of the 134 eligible patients, 66 received standard-dose radiotherapy and 68 received high-dose radiotherapy. Baseline characteristics of the patients are shown in Table 1. Compared to those who received high-dose radiotherapy, patients who received standard-dose radiotherapy were older (*p* = 0.010) and underwent pretreatment staging combined with endoscopic ultrasonography, IMRT/VMAT, and ENI more often (*p* <  0.001, *p* = 0.007, and *p* <  0.001, respectively). In the subsequent analyses, we did not include the use of endoscopic ultrasonography, radiotherapy techniques, or ENI as covariates, because of the non-adjustable differences between the two groups. The standardized mean differences of unweighted comparisons significantly differed in all covariates, except for chemotherapy regimen (Additional file 2). The distributions of estimated PSs, ROC curve, and c-statistic are shown in Additional file 3. After PS weighting, the standardized mean difference for all covariates was confirmed to be < 0.10, indicating that the distribution of all covariates was adequately balanced (Additional file 2).

**Table 1:** Patient characteristics stratified by treatment group

Characteristic	Overall (*n* = 134)	Standard-dose (*n* = 66)	High-dose (*n* = 68)	*P*-value
Age, years				0.010
Median (IQR)	67.0 (62.0–74.0)	70.0 (63.0–76.0)	65.5 (61.0–69.2)	
Range	40.0–86.0	40.0–86.0	47.0–79.0	
Sex, n (%)				0.51
Female	20 (14.9)	8 (12.1)	12 (17.6)	
Male	114 (85.1)	58 (87.9)	56 (82.4)	
Tumor location, n (%)				0.68
Cervical esophagus	6 (4.5)	3 (4.5)	3 (4.4)	
Upper thoracic esophagus	18 (13.4)	9 (13.6)	9 (13.2)	
Middle thoracic esophagus	67 (50.0)	36 (54.5)	31 (45.6)	
Lower thoracic esophagus	43 (32.1)	18 (27.3)	25 (36.8)	
Tumor length, mm				0.62
Median (IQR)	45.0 (30.0–70.0)	45.0 (30.0–77.5)	45.0 (30.0–70.0)	
Range	4.0–180.0	5.0–180.0	4.0–150.0	
Clinical depth of invasion, n (%)				0.51
EP/LPM	33 (24.6)	19 (28.8)	14 (20.6)	
MM/SM1	32 (23.9)	14 (21.2)	18 (26.5)	
SM2	69 (51.5)	33 (50.0)	36 (52.9)	
Pretreatment staging combined with EUS				
No	100 (74.6)	39 (59.1)	61 (89.7)	< 0.001
Yes	34 (25.4)	27 (40.9)	7 (10.3)	
Radiotherapy technique, n (%)				0.007
3D-CRT	118 (88.1)	53 (80.3)	65 (95.6)	
IMRT/VMAT	16 (11.9)	13 (19.7)	3 (4.4)	
Elective nodal irradiation, n (%)				< 0.001
No	68 (50.7)	9 (13.6)	59 (86.8)	
Yes	66 (49.3)	57 (86.4)	9 (13.2)	
Chemotherapy, n (%)				1.000
Cisplatin + 5-fluorouracil	124 (92.5)	61 (92.4)	63 (92.6)	
Others	10 (7.5)	5 (7.6)	5 (7.4)	

*Abbreviations*: *IQR* Interquartile range, *EP/LPM* Tumor limited to the epithelium or invading the lamina propria mucosa, *MM/SM1* Tumor invading the muscularis mucosa or submucosa to a depth of ≤ 200 μm from the lower border of the muscularis mucosa, *SM2* Tumor invading the submucosa to a depth of > 200 μm, *EUS* Endoscopic ultrasonography, *3D-CRT* Three-dimensional conformal radiotherapy, *IMRT/VMAT* Intensity-modulated radiotherapy/volumetric modulated arc therapy

### Outcomes

**Fig. 1:**
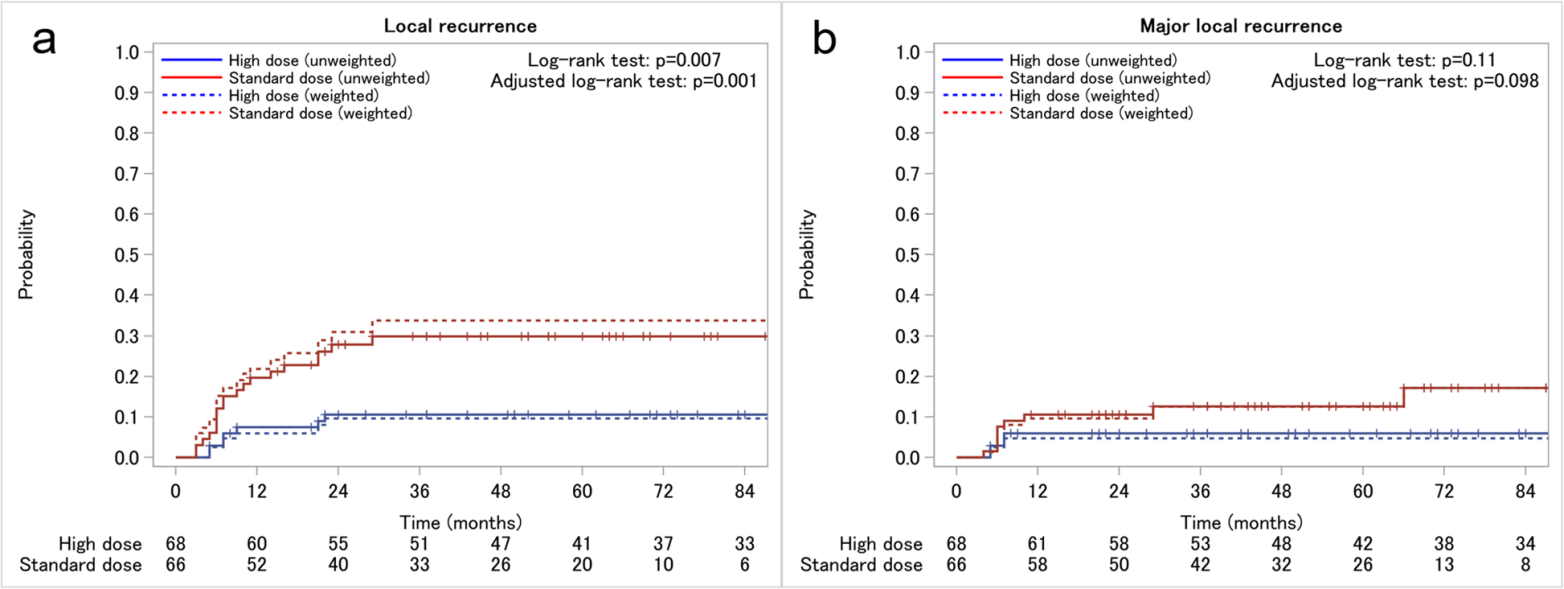
Local recurrence (**a**) and major local recurrence (**b**) curves of the treatment groups. The curves are based on values obtained with and without propensity score weighting. Number at risk is calculated from the unweighted population

**Fig. 2:**
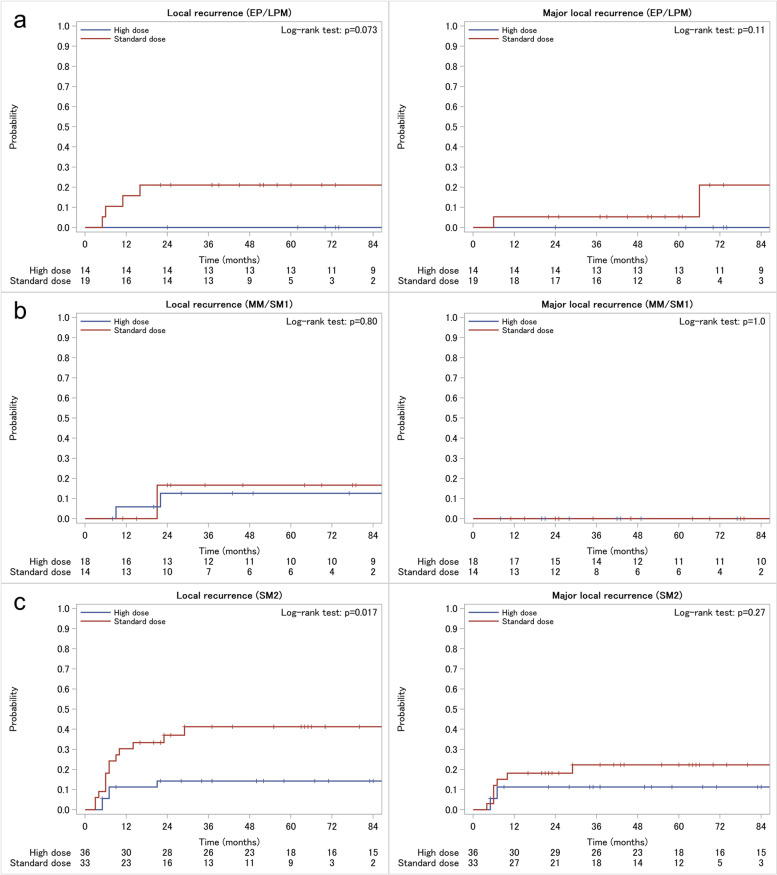
Local recurrence (left) and major local recurrence (right) curves. Curves are stratified by clinical depth of invasion: **a** EP/LPM (tumors limited to the epithelium or invading the lamina propria mucosa), **b** MM/SM1 (tumors invading the muscularis mucosa or the submucosa to a depth of ≤ 200 μm from the lower border of the muscularis mucosa), and **c** SM2 (tumors invading the submucosa to a depth of > 200 μm)

The median follow-up periods were 52 (interquartile range [IQR], 25–70) and 84 (IQR, 37–126) months in the standard-dose and high-dose groups, respectively. The 3-year overall survival rates were 79.3 and 80.8% in the standard-dose and high-dose groups, respectively. LR and MLR occurred in 19 (29%) and nine (14%) patients in the standard-dose group and seven (10%) and four (5.9%) patients in the high-dose group, respectively. In the IPTW-adjusted Kaplan–Meier analysis (Fig. 1), the standard-dose group had a higher incidence of LR (adjusted HR, 4.00 [95% confidence interval [CI]: 1.64–9.73]; adjusted log-rank *p* = 0.001) (Fig. 1a) and MLR (adjusted HR, 3.13 [95% CI: 0.91–10.81]; adjusted log-rank *p* = 0.098) (Fig. 1b) than the high-dose group, although the difference in MLR was not statistically significant. The adjusted 3-year LR and MLR rates were 33.8% (95% CI: 23.2–47.5) and 12.5% (95% CI: 6.1–24.8) in the standard-dose group and 9.6% (95% CI: 4.3–20.7) and 4.7% (95% CI: 1.5–14.3) in the high-dose group, respectively. In the Kaplan–Meier analysis stratified by clinical DOI (Fig. 2), the standard-dose group had a higher incidence of 3-year LR in the EP/LPM (21.1% [95% CI: 8.5–46.8] vs. 0.0%) and SM2 (41.2% [95% CI: 26.2–60.6] vs. 14.2% [95% CI: 6.2–30.9]) subgroups than the high-dose group. In MLR analysis, these observed differences decreased in the EP/LPM (5.3% [95% CI: 0.8–31.9] vs. 0.0%) and SM2 (22.3% [95% CI: 11.2–41.4] vs. 11.3% [95% CI: 4.4–27.3]) subgroups. In the MM/SM1 subgroup, the difference in LR and MLR between the two groups was small (3-year LR: 16.7% [95% CI: 4.4–51.8] vs. 12.6% [95% CI: 3.3–41.9]; 3-year MLR: 0.0% vs. 0.0%).

In the univariable and multivariable Cox regression analyses (Tables 2 and 3), clinical DOI was stratified into two groups (EP/LPM and MM/SM1 vs. SM2), because no MLR was observed in the MM/SM1 subgroup. Standard-dose radiotherapy was independently associated with a higher incidence of LR (HR, 4.09 [95% CI: 1.65–10.14]; *p* = 0.002) and MLR (HR, 4.00 [95% CI: 1.13–14.17]; *p* = 0.031) than high-dose radiotherapy. The clinical DOI of SM2 was an independent predictor of LR (HR, 2.89 [95% CI: 1.14–7.34]; *p* = 0.026) and MLR (HR, 8.17 [95% CI: 1.45–45.91]; *p* = 0.017).

**Table 2:** Univariable and multivariable analyses of local recurrence

Covariates	Univariable analysis			Multivariable analysis		
	HR	CI	*p*-value	HR	CI	*p*-value
Dose group						
High-dose (reference)	1.00	–	–	1.00	–	–
Standard-dose	3.08	1.30–7.34	0.011	4.09	1.65–10.14	0.002
Age (years)						
40–64 (reference)	1.00	–	–	–	–	–
65–74	0.94	0.39–2.26	0.88	–	–	–
75–86	0.87	0.32–2.36	0.79	–	–	–
Sex						
Female (reference)	1.00	–	–	–	–	–
Male	0.96	0.33–2.77	0.93	–	–	–
Tumor location						
Mt (reference)	1.00	–	–	1.00	–	–
Ce or Ut	2.34	0.87–6.29	0.092	3.39	1.17–9.78	0.024
Lt	1.89	0.77–4.66	0.16	3.00	1.13–7.93	0.027
Tumor length (mm)						
≤ 40 (reference)	1.00	–	–	1.00	–	–
> 40 and ≤ 80	0.33	0.11–0.99	0.048	0.42	0.14–1.28	0.13
> 80	1.18	0.46–3.02	0.73	2.72	0.90–8.21	0.075
Clinical depth of invasion						
EP/LPM or MM/SM1 (reference)	1.00	–	–	1.00	–	–
SM2	2.37	1.03–5.46	0.042	2.89	1.14–7.34	0.026
Chemotherapy						
Cisplatin + 5-fluorouracil (reference)	1.00	–	–	–	–	–
Others	1.83	0.55–6.08	0.33	–	–	–

*Abbreviations*: *HR* Hazard ratio, *CI* Confidence interval, *Mt* Middle thoracic esophagus, *Ce* Cervical esophagus, *Ut* Upper thoracic esophagus, *Lt* Lower thoracic esophagus, *EP/LPM* Tumor limited to the epithelium or invading the lamina propria mucosa, *MM/SM1* Tumor invading the muscularis mucosa or submucosa to a depth of ≤ 200 μm from the lower border of the muscularis mucosa, *SM2* Tumor invading the submucosa to a depth of > 200 μm

**Table 3:** Univariable and multivariable analyses for major local recurrence

Covariates	Univariable analysis			Multivariable analysis		
	HR	CI	*p*-value	HR	CI	*p*-value
Dose group						
High-dose (reference)	1.00	–	–	1.00	–	–
Standard-dose	2.50	0.77–8.17	0.13	4.00	1.13–14.17	0.031
Age (years)						
40–64 (reference)	1.00	–	–	–	–	–
65–74	0.77	0.22–2.74	0.69	–	–	–
75–86	0.85	0.21–3.41	0.82	–	–	–
Sex						
Female (reference)	1.00	–	–	–	–	–
Male	0.98	0.22–4.43	0.98	–	–	–
Tumor location						
Mt (reference)	1.00	–	–	1.00	–	–
Ce or Ut	3.00	0.75–12.04	0.12	4.24	0.91–19.80	0.066
Lt	1.97	0.53–7.35	0.31	2.86	0.70–11.59	0.14
Tumor length (mm)						
≤ 40 (reference)	1.00	–	–	1.00	–	–
> 40 and ≤ 80	0.15	0.019–1.20	0.074	0.25	0.03–2.01	0.19
> 80	1.11	0.30–4.09	0.88	4.5	0.93–21.91	0.062
Clinical depth of invasion						
EP/LPM or MM/SM1 (reference)	1.00	–	–	1.00	–	–
SM2	5.67	1.26–25.62	0.024	8.17	1.45–45.91	0.017
Chemotherapy						
Cisplatin + 5-fluorouracil (reference)	1.00	–	–	1.00	–	–
Others	2.82	0.62–12.88	0.18	3.24	0.66–15.87	0.15

*Abbreviations*: *HR* Hazard ratio, *CI* Confidence interval, *Mt* Middle thoracic esophagus, *Ce* Cervical esophagus, *Ut* Upper thoracic esophagus, *Lt* Lower thoracic esophagus, *EP/LPM* Tumor limited to the epithelium or invading the lamina propria mucosa, *MM/SM1* Tumor invading the muscularis mucosa or submucosa to a depth of ≤ 200 μm from the lower border of the muscularis mucosa, *SM2* Tumor invading the submucosa to a depth of > 200 μm

## Discussion

Our study revealed that standard-dose radiotherapy is associated with a higher incidence of LR and MLR than high-dose radiotherapy. Although several studies have investigated the benefits of brachytherapy boost following external beam radiotherapy [13–15], to our best knowledge, this is the first study to directly compare the effects of standard-dose vs. high-dose radiotherapy on local tumor control in patients with non-metastatic SESCC treated with concurrent chemoradiotherapy. Our subgroup analyses indicated that salvage endoscopic resection can reduce differences in MLR between the two dose groups in the EP/LPM and MM/SM1 subgroups, although noticeable differences may be apparent in the SM2 subgroup. Univariable and multivariable analyses revealed that the clinical DOI of SM2 is a strong predictor of MLR. As endoscopic resection is generally indicated for mucosal or shallow submucosal tumors [3, 16], it is reasonable to assume that there would be considerable difficulty in performing salvage endoscopic resection for recurrent lesions that initially had deep submucosal invasion (such as SM2 tumors). Salvage esophagectomy may be proposed for LR that cannot be eliminated by endoscopic resection; however, esophagectomy following chemoradiotherapy may lead to high rates of morbidity and mortality [17, 18]. In this context, we believe that when careful follow-ups with periodic endoscopy and salvage endoscopic resection are feasible, both standard-dose and high-dose radiotherapy are appropriate treatments for EP/LPM and MM/SM1 tumors, whereas SM2 tumors may require high-dose radiotherapy.

Interestingly, our study indicated that the incidence of LR in the standard-dose group, compared to that in the high-dose group, may be high, even in the EP/LPM subgroup, although the reason is unclear. A possible explanation for this is the difference in tumor length between the two groups. There were more patients with EP/LPM tumors with a tumor length > 80 mm in the standard-dose group than in the high-dose group. In fact, several studies have reported tumor length as a predictor of LR or locoregional recurrence [19, 20].

This study was limited by its retrospective study design and small sample size. Our study included patients who were treated between 2006 and 2019. During this extended period, there were several changes in chemotherapy regimens for recurrence and radiotherapy techniques. In this study, we did not compare the rates of overall survival and adverse events between the treatment groups due to the difficulty of accounting for these changes. Currently, the results of an ongoing trial comparing standard-dose and high-dose radiotherapy for clinical T1bN0M0 esophageal cancer in Japan (JCOG 1904; ClinicalTrials.gov Identifier: NCT04328948) are awaited. We performed IPTW and univariable and multivariable analyses to adjust for differences in baseline characteristics between the treatment groups; however, some covariates that may affect the outcomes—including radiotherapy techniques, treatment era, and the use of pretreatment endoscopic ultrasonography—could not be adjusted for. The use of endoscopic resection for larger or deeper SESCCs has been recently challenged at our institution [9], and patients treated with definitive chemoradiotherapy in the modern era may have had more extensive or deeper SESCCs. To account for this change, we included clinical DOI and tumor length as covariates. However, our results may be potentially affected by biases from unobserved differences. Although univariable and multivariable analyses revealed the significant benefit of high-dose radiotherapy for MLR, the IPTW analysis did not show statistically significant differences in MLR.

## Conclusions

Our study revealed that high-dose radiotherapy had more positive effects on local tumor control than standard-dose radiotherapy in patients with non-metastatic SESCC who underwent definitive chemoradiotherapy. We also found that the clinical DOI of SM2 tumors is a strong predictor of recurrence that cannot be eliminated by endoscopic resection. The use of high-dose radiotherapy may merit consideration, especially for the treatment of SM2 tumors. Further prospective studies comparing patient outcomes, including survival and adverse events, are needed to determine the optimal dose for patients with non-metastatic SESCC.

## Supplementary Information

**Additional file 1.** Other chemotherapy regimens.

**Additional file 2.** Patient characteristics of the treatment groups with standardized mean differences before and after propensity score weighting.

**Additional file 3.** Propensity scores of the standard-dose and high-dose groups and the receiver operating characteristic curve.

**Additional file 4.** Dataset used in this study.

## Data Availability

The dataset used in the current study is available in the supporting information files. The raw dataset is available from the corresponding author upon reasonable request as it includes some indirect identifiers.

## Abbreviations

CTV: Clinical target volume
DOI: Depth of invasion
ENI: Elective nodal irradiation
HR: Hazard ratio
IMRT/VMAT: Intensity-modulated radiotherapy/volumetric modulated arc therapy
IPTW: Inverse probability of treatment weighting
IQR: Interquartile range
LR: Local recurrence
MLR: Major local recurrence
PS: Propensity score
ROC: Receiver operating characteristic
SESCC: Superficial esophageal squamous cell carcinoma
