# Supplementary material for: Influence of radiation dose and predicted tumor invasion depth on local recurrence after definitive chemoradiotherapy for stage 0–I esophageal squamous cell carcinoma: a propensity score-weighted, retrospective, observational study
Source: BMC Cancer. 2022 Mar 21;22:301. doi: 10.1186/s12885-022-09418-2 (PMC8939113; doi:10.1186/s12885-022-09418-2)
Supplement: Supplementary file 1 — Additional file 1. Other chemotherapy regimens. [file 12885_2022_9418_MOESM1_ESM.docx]

**Additional File 1.** Other chemotherapy regimens

| Nedaplatin (90 mg/m^2^/day on Days 1 and 36) + 5-fluorouracil (800 mg/m^2^/day as a continuous infusion on Days 1–5 and 36–40) |
| --- |
| Docetaxel (10 mg/m^2^/day weekly) |
| 5-fluorouracil alone (700 mg/m^2^/day as a continuous infusion on Days 1–4 and 29–32) |
