# Supplementary material for: Influence of radiation dose and predicted tumor invasion depth on local recurrence after definitive chemoradiotherapy for stage 0–I esophageal squamous cell carcinoma: a propensity score-weighted, retrospective, observational study
Source: BMC Cancer. 2022 Mar 21;22:301. doi: 10.1186/s12885-022-09418-2 (PMC8939113; doi:10.1186/s12885-022-09418-2)
Supplement: Supplementary file 2 — Additional file 2. Patient characteristics of the treatment groups with standardized mean differences before and after propensity score weighting. [file 12885_2022_9418_MOESM2_ESM.docx]

**Additional File 2.** Patient characteristics of the treatment groups with standardized mean differences before and after propensity score weighting

| Characteristic | Unweighted, n (%) | |  | Propensity score-weighted, % | |  | Standardized mean difference | |
| --- | --- | --- | --- | --- | --- | --- | --- | --- |
|  | High-dose (n = 68) | Standard-dose (n = 66) |  | High-dose | Standard-dose |  | Unweighted | Propensity score-weighted |
| Age (years) |  |  |  |  |  |  | 0.43 | 0.014 |
| 40–64 | 32 (47.1) | 21 (31.8) |  | 40.6 | 41.3 |  |  |  |
| 65–74 | 25 (36.8) | 23 (34.8) |  | 34.5 | 34.1 |  |  |  |
| 75–86 | 11 (16.2) | 22 (33.3) |  | 24.9 | 24.6 |  |  |  |
| Sex |  |  |  |  |  |  | 0.16 | 0.024 |
| Female | 12 (17.6) | 8 (12.1) |  | 14.2 | 15.0 |  |  |  |
| Male | 56 (82.4) | 58 (87.9) |  | 85.8 | 85.0 |  |  |  |
| Tumor location |  |  |  |  |  |  | 0.21 | 0.026 |
| Ce or Ut | 12 (17.6) | 12 (18.2) |  | 19.3 | 18.3 |  |  |  |
| Mt | 31 (45.6) | 36 (54.5) |  | 47.4 | 48.1 |  |  |  |
| Lt | 25 (36.8) | 18 (27.3) |  | 33.2 | 33.5 |  |  |  |
| Tumor length (mm) |  |  |  |  |  |  | 0.24 | 0.053 |
| ≤ 40 | 34 (50.0) | 33 (50.0) |  | 50.8 | 51.8 |  |  |  |
| > 40 and ≤ 80 | 26 (38.2) | 20 (30.3) |  | 36.3 | 34.1 |  |  |  |
| > 80 | 8 (11.8) | 13 (19.7) |  | 12.9 | 14.1 |  |  |  |
| Clinical depth of invasion |  |  |  |  |  |  | 0.2 | 0.024 |
| EP/LPM | 14 (20.6) | 19 (28.8) |  | 24.6 | 24.5 |  |  |  |
| MM/SM1 | 18 (26.5) | 14 (21.2) |  | 23.2 | 22.3 |  |  |  |
| SM2 | 36 (52.9) | 33 (50.0) |  | 52.2 | 53.1 |  |  |  |
| Chemotherapy |  |  |  |  |  |  | 0.01 | 0.019 |
| Cisplatin + 5-fluorouracil | 63 (92.6) | 61 (92.4) |  | 92.1 | 92.6 |  |  |  |
| Others | 5 (7.4) | 5 (7.6) |  | 7.9 | 7.4 |  |  |  |

Abbreviations: Ce, cervical esophagus; Ut, upper thoracic esophagus; Mt, middle thoracic esophagus; Lt, lower thoracic esophagus; EP/LPM, tumor limited to the epithelium or invading the lamina propria mucosa; MM/SM1, tumor invading the muscularis mucosa or submucosa to a depth of ≤ 200 μm from the lower border of the muscularis mucosa; SM2, tumor invading the submucosa to a depth of > 200 μm.
