# Supplementary material for: Influence of radiation dose and predicted tumor invasion depth on local recurrence after definitive chemoradiotherapy for stage 0–I esophageal squamous cell carcinoma: a propensity score-weighted, retrospective, observational study
Source: BMC Cancer. 2022 Mar 21;22:301. doi: 10.1186/s12885-022-09418-2 (PMC8939113; doi:10.1186/s12885-022-09418-2)
Supplement: Supplementary file 3 — Additional file 3. Propensity scores of the standard-dose and high-dose groups and the receiver operating characteristic curve. [file 12885_2022_9418_MOESM3_ESM.docx]

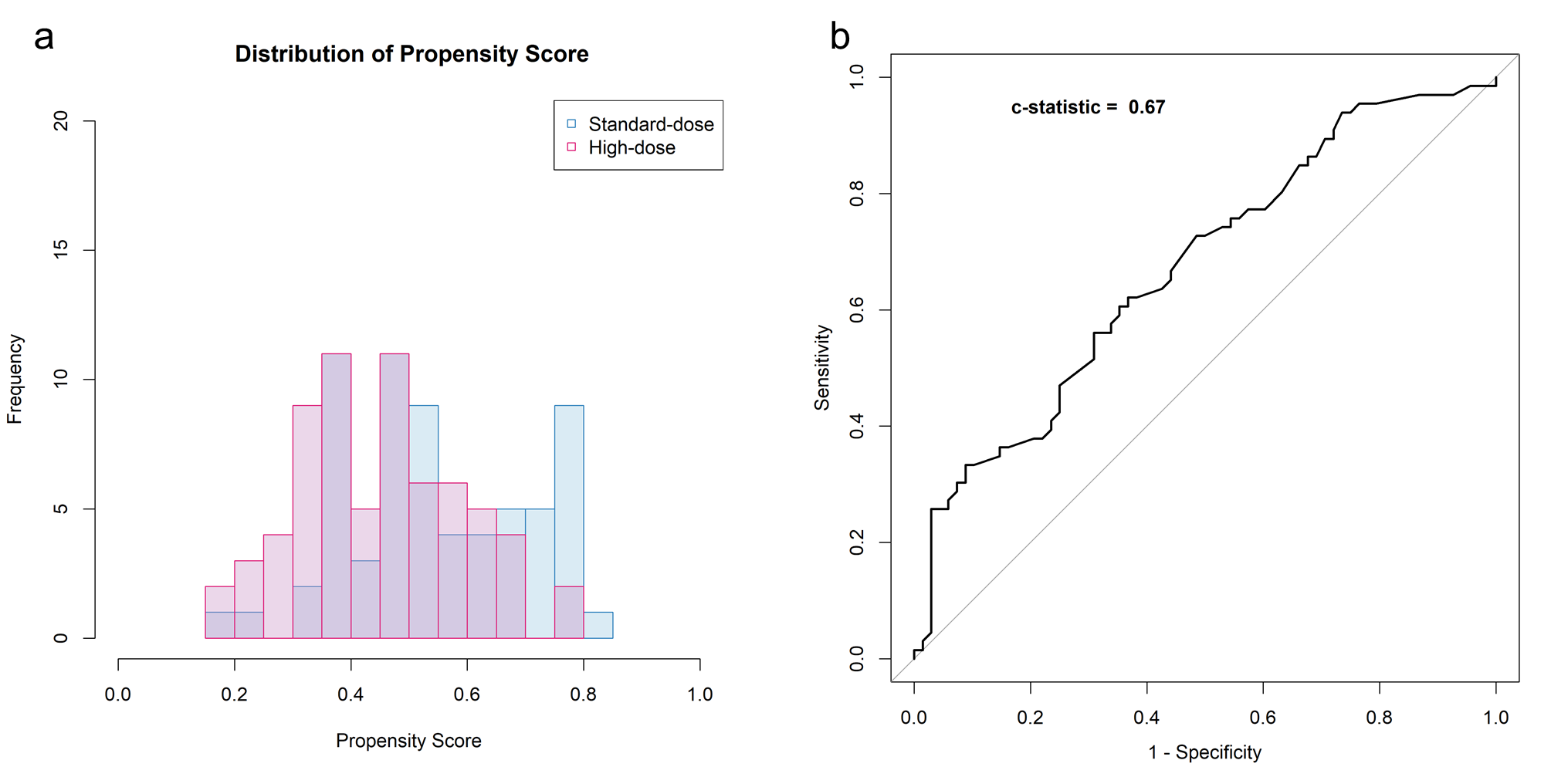


**Additional File 3.** Propensity scores of the standard-dose and high-dose groups and the receiver operating characteristic curve. **(a)** Distribution of propensity scores between the standard-dose and high-dose treatment groups. **(b)** Receiver operating characteristic curve discriminating between the two groups (c-statistic: 0.67).
